# Growth of carbon nanowalls at atmospheric pressure for one-step gas sensor fabrication

**DOI:** 10.1186/1556-276X-6-202

**Published:** 2011-03-09

**Authors:** Kehan Yu, Zheng Bo, Ganhua Lu, Shun Mao, Shumao Cui, Yanwu Zhu, Xinqi Chen, Rodney S Ruoff, Junhong Chen

**Affiliations:** 1 Department of Mechanical Engineering, University of Wisconsin-Milwaukee, Milwaukee, WI 53211, USA; 2 Department of Mechanical Engineering and the Texas Materials Institute, University of Texas at Austin, Austin, TX 78712, USA; 3 Keck-II Center, Northwestern University, Evanston, IL 60208, USA

## Abstract

Carbon nanowalls (CNWs), two-dimensional "graphitic" platelets that are typically oriented vertically on a substrate, can exhibit similar properties as graphene. Growth of CNWs reported to date was exclusively carried out at a low pressure. Here, we report on the synthesis of CNWs at atmosphere pressure using "direct current plasma-enhanced chemical vapor deposition" by taking advantage of the high electric field generated in a pin-plate dc glow discharge. CNWs were grown on silicon, stainless steel, and copper substrates without deliberate introduction of catalysts. The as-grown CNW material was mainly mono- and few-layer graphene having patches of O-containing functional groups. However, Raman and X-ray photoelectron spectroscopies confirmed that most of the oxygen groups could be removed by thermal annealing. A gas-sensing device based on such CNWs was fabricated on metal electrodes through direct growth. The sensor responded to relatively low concentrations of NO_2 _(g) and NH_3 _(g), thus suggesting high-quality CNWs that are useful for room temperature gas sensors.

PACS: Graphene (81.05.ue), Chemical vapor deposition (81.15.Gh), Gas sensors (07.07.Df), Atmospheric pressure (92.60.hv)

## Introduction

Graphene possesses many extraordinary properties and has been the subject of intense scientific interest [1-12]. Exceptional values have been reported of: ballistic electron mobility (>200,000 cm^2^/V-s for particular samples) [13,14], high thermal conductivity (5,000 W/m-K) [15], Young's modulus (approximately 1,100 GPa), fracture strength (125 GPa) [16], and a high specific surface area (approximately 2,600 m^2^/g) relevant to electrical energy storage [5].

"Carbon nanowalls" (CNWs), also referred to as "carbon nanoflakes", are two-dimensional "graphitic" platelets that are typically oriented vertically on a substrate. An individual CNW has been reported to have a few stacked layers ("graphitic") with typical lateral dimensions of several micrometers [17]. CNWs might exhibit similar properties as graphene. The sharp edges and vertical orientation make CNWs a potential field emission material [18-20]. The high surface area of CNWs could be ideal for catalyst support. Recently, CNWs have been tested for use in Li-ion batteries [21] and electrochemical capacitors [22]. CNWs can also be used as a template for loading other nanomaterials; and the resulting hybrid nanostructures are potentially useful for various applications [23-25].

CNWs were discovered by Wu et al. [26] and since then they have been grown using various low-pressure processes. Initially, substrates were sputter-coated with transition metals as catalysts and the growth of CNWs was typically carried out in a microwave plasma-enhanced chemical vapor deposition (MPECVD) system [23]. Only a few studies of CNW growth using low-pressure, low-voltage, high-current dc PECVD have been conducted [27]. The growth parameters were very similar to those used for PECVD growth of carbon nanotubes (CNTs), but the pressure used in the reactor chamber was much lower (≤1 Torr) [17,26-31]. There have been a number of studies focused on understanding the CNW growth mechanism and thus targeting control of the growth process [22,26,32,33]. Nevertheless, to our knowledge, no CNW growth has been reported at atmospheric pressure.

Here, we report on the synthesis of CNWs using dc PECVD at atmospheric pressure by taking advantage of the high electric field generated in a pin-plate dc glow discharge. In general, PECVD processes for the material growth can occur at a relatively lower temperature due to the significant contribution from energetic electrons to cracking down precursor species. Prior studies using low-pressure PECVD systems to grow CNWs mainly rely on the increased mean free path (mfp) of electrons in vacuum to obtain energetic electrons needed for the decomposition of carbon precursors. The electric field generated in the low-pressure PECVD system is generally low. By using a pair of asymmetric discharge electrodes, i.e., a sharpened tungsten tip as cathode and a planar substrate as anode, a highly enhanced electric field about two to three orders of magnitude higher than that in the previous MPECVD system is generated near the tungsten tip so that the mfp of electrons can be lowered or the system pressure can be elevated (e.g., to atmospheric pressure) to generate similar energetic electrons.

Our method does not require a sealed reactor, which presents a path for continuous line production of CNWs. An atmospheric-pressure process to replace the vacuum process should also reduce the product cost. A recent study on the high cost of modern vacuum deposition methods highlighted the need for atmospheric synthesis [34]. The as-grown CNWs were decorated with oxygen-containing functional groups. By thermal annealing in H_2_, most oxygen functional groups can be effectively eliminated. In addition, most of the product CNWs are non-aggregated with large surface area, which makes the product readily useful for various applications such as sensing and catalysis. This is in contrast to stacked CNWs that require additional dispersion, such as through ultrasonication, to obtain individual CNWs. To illustrate the advantage of our growth method, CNWs deliberately grown between metal electrodes were used for detection of low-concentration gases including NO_2 _and NH_3_, thereby demonstrating a one-step gas sensor fabrication process.

## Experimental details

The plasma reactor consists of a quartz tube that houses a tungsten needle cathode, a grounded graphite rod anode, and a dc high negative voltage supply (EMCO 4100N; up to -10 kV) to drive the dc glow discharge. Argon was used as the plasma gas. A tube furnace (TF55035 A-1, Lindberg/BLUE M, Asheville, USA) was used to heat the reactor. Silicon wafers, stainless steel plates, and Cu plates were used as substrates. The substrates were mounted on the top of the graphite rod; no metals were added as potential catalysts.

Prior to the growth, the substrate was brought to 700°C and held at that temperature for 10 min in an Ar/H_2 _flow (1% H_2 _by volume) of 500 standard cubic centimeters per minute (sccm). The two discharge electrodes were separated by a distance of 1.0 cm. Then the Ar/H_2 _flow was switched to an Ar/ethanol flow (1,000 sccm) through an ethanol bubbler. The dc glow discharge was ignited at a dc voltage of 3.3 kV. Once the dc plasma was formed, the voltage between the electrodes immediately dropped to 2.2 kV, and the current was about 1.3 mA, yielding a total plasma power of 2.9 W.

The plasma was typically left on for 15 min. Then, the plasma was turned off and the system was cooled down to room temperature with a flow of Ar/H_2 _only. Throughout the process, the reactor pressure was maintained at one atmosphere. The reactor temperature was measured as close to 700°C (the preset furnace temperature) using a thermocouple. This suggests that the energy dissipated in the dc glow discharge was non-thermal (electrons were preferentially heated by the plasma) and heavy species (e.g., gas molecules, atoms, radicals, and ions) were not substantially heated by the plasma. After the plasma was turned off, a layer of black, powder-like material could be seen on the substrate. In order to reduce oxygen functional groups decorated on the as-grown CNWs, the CNWs were thermally annealed at 900°C in H_2 _flow (1,000 sccm) for 2 h at atmospheric pressure.

Scanning electron microscopy (SEM) analysis of the as-grown samples was performed with a Hitachi S-4800 SEM having a stated resolution of 1.4 nm at 1 kV acceleration voltage. Transmission electron microscopy (TEM) was performed with a Hitachi H 9000 NAR TEM, which has a stated point resolution of 0.18 nm at 300 kV in the phase contrast, high-resolution TEM (HRTEM) imaging mode. In order to perform TEM characterizations, the as-grown CNWs were wetted with ethanol and contact-transferred to lacey carbon-coated TEM grids or bare Cu grids. A confocal Raman system, which is composed of a TRIAX 320 spectrograph, liquid nitrogen-cooled CCD (CCD 3000), and "spectrum one" CCD controller (all manufactured by HORIBA Jobin Yvon), was used to record the Raman spectra of the samples with an excitation wavelength of 532 nm. X-ray photoelectron spectroscopy (XPS, Omicron NanoESCA probe, Omicron NanoTechnology GmbH, Taunusstein, Germany) was used to analyze the chemical composition as well as the nature of the chemical bonds in the as-grown CNWs.

Gold-interdigitated electrodes with both finger width and inter-finger spacing of about 1 μm and thickness of 50 nm were fabricated using an e-beam lithography process (Raith 150 lithography tool, 30 kV) on an Si wafer with a top layer of thermally-formed SiO_2 _(thickness of 200 nm). Sensor current was measured using a Keithley 2602 source meter.

## Results and discussion

**Figure 1:**
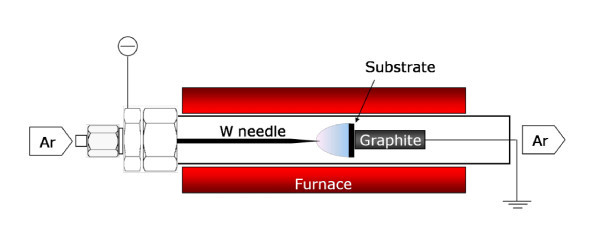
**Experimental setup for atmospheric pressure dc PECVD growth of CNWs**.

**Figure 2:**
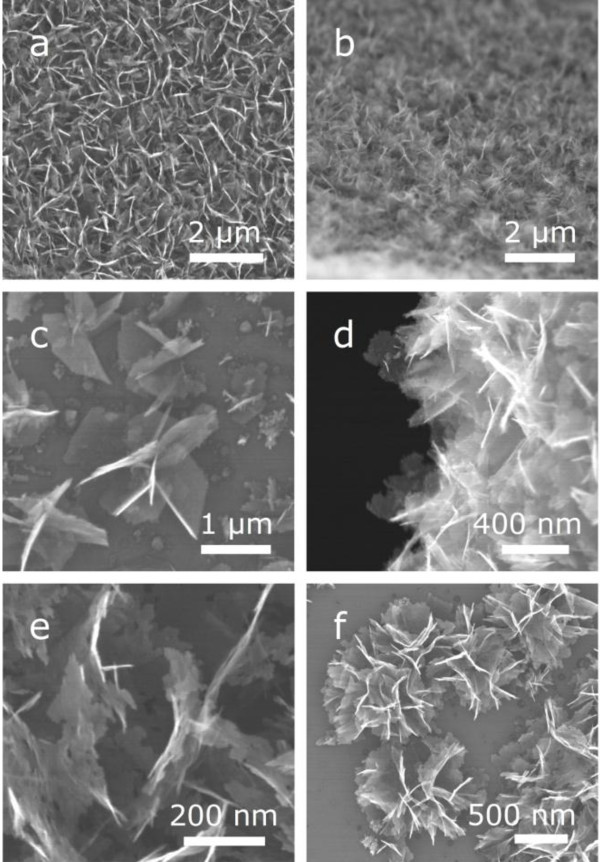
**Morphology of the as-grown CNWs displayed in the SEM images**. **(a) **An SEM image of CNWs on a silicon substrate; primary beam incident kinetic energy was 30 keV. **(b) **CNWs uniformly distributed on the substrate over approximately 1 cm^2^. **(c-e) **The CNWs were quasi-transparent to the SEM electron beam. **(f) **The cluster of CNWs is "flower-like".

Figure 1 shows a schematic of the atmospheric dc PECVD system for the CNW synthesis without any catalysts. The morphology of the as-grown CNWs is displayed in the SEM images shown in Figure 2. The CNWs were uniformly distributed on the Si substrate (Figure 2a,2b). The total area on the substrate that was covered with CNWs depended on the discharge power and the distance between the electrodes. In our experiments, the area covered with CNWs could be up to approximately 1 cm^2^. The dimensions of individual CNWs ranged from about 200 × 200 nm^2 ^(Figure 2e) to 1 × 1 μm^2 ^(Figure 2c), which can be controlled by the growth time. The thickness of the CNWs was typically below 10 nm, (top-view of CNWs, Figure 2c,2e; side-view of CNWs, Figure 2d). Small pinholes were observed in the CNWs (Figure 2e). Wu et al. used a dc bias of -185 V to promote growth and vertical alignment [26]. Hiramatsu et al. stated that the reactant type influences the CNW morphology [30], in the case of C_2_F_6_/H_2_, they synthesized vertically aligned CNWs using a radio-frequency plasma. In our experiments, most of the CNWs were randomly oriented but pointing away from the substrate surface, although a dc bias of 2.2 kV was applied between the electrodes throughout the growth process. In some areas, CNW clusters were found (Figure 2f) sparsely distributed on the substrate. Each CNW cluster had a "flower-like" shape with CNWs projecting in all directions, which is similar to the observations made by Chuang et al [35]. Similar structures were also found for CNWs grown on a Cu substrate (see Figure S-1 in Additional file 1).

**Figure 3:**
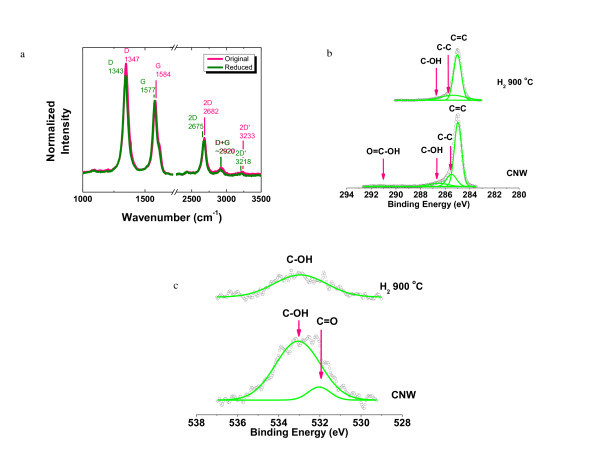
**Raman and XPS spectra**. **(a) **Raman spectrum of CNWs (original and reduced) showing the presence of D and G bands as well as the overtone and combination mode features taken with 532 nm laser excitation. **(b) **The C1 s and **(c) **the O1 s XPS spectra of CNWs before and after thermal annealing. The as-grown CNWs contained many oxygen functional groups, while only a low fraction of hydroxyl groups remained after thermal reduction in H_2 _for 2 h at 900°C. The peak components (green curves) were analyzed with a Gaussian fit.

Raman spectra showed D and G bands located at 1,347 and 1,584 cm^-1^, respectively (Figure 3a). The bulk graphite has a G peak at approximately 1,580 cm^-1 ^[36], whereas a D peak at approximately 1,350 cm^-1 ^is seen for defective graphite [37]. The position and shape of the G peak suggest that graphitized carbon was synthesized. The 2 D band (2,682 cm^-1^) suggests the presence of "graphene-like" materials. A very small 2D' band (approximately 3,233 cm^-1^) indicates the existence of the D' band that is however probably convoluted with the G band. The G peak for graphene sheets [38,39] occurs at approximately 1,580 cm^-1^, and this peak broadens and significantly shifts to 1,594 cm^-1 ^for graphite oxide sheets [40,41]. The upshift of what we attribute as the G peak (to 1,584 cm^-1^) suggests a possibility of a high fraction of oxygen contained in the as-grown CNWs. In the growth of CNTs, it was stated that oxygen etches the carbon on the catalyst particle surface and thus promotes CNT growth [42]. We found that oxygen-containing radicals also appear to be essential for the growth of CNWs in our growth attempts. Hung et al. attributed the formation of nucleation sites for the growth of CNWs to the etching by oxygen-containing species [22]. In addition to using ethanol, we tried to synthesize CNWs with pure CH_4 _or with n-hexane vapor with Ar as the carrier gas, but no CNWs were observed. However, CNWs could be readily synthesized with CH_4 _and water vapor (again with Ar as the carrier gas), where the presence of C-OH groups was confirmed with optical emission spectroscopy (see Figure S-2 and S-3 in Additional file 1). The 1:2 O/C ratio in the ethanol precursor is perhaps too high to produce high-purity "graphene-like" material with the approach we have used, but we note the recent report of very carbon-pure graphene made from ethanol using a microwave plasma operated at low pressure [43]. It is likely that the oxygen radicals etch away carbon as it is deposited during the growth, which may explain broken edges and pinholes on the resulting CNW sheets.

The 2 D peak is a signature of graphitic carbon in the graphene-like materials [11]. The Raman spectrum obtained from the as-grown CNWs exhibits a peak centered at 2,682 cm^-1 ^(Figure 3a, pink curve), indicating that the analyzed region consists of considerable amount of graphene or oxygenated graphene. After thermal annealing, the 2 D peak shifted to 2,675 cm^-1 ^(Figure 3a, olive curve). This trend is in agreement with literature. The 2 D peaks were reported at 2,861 cm^-1 ^for monolayer graphene oxide [44], and 2,700 cm^-1 ^for monolayer graphene [45]. For monolayer reduced graphene oxide, the 2 D peak was found around 2,700 cm^-1 ^or below 2,700 cm^-1 ^[44,46,47]. The 2 D band is very sensitive to the number of layers in the sample. Figure 3a shows single Lorentzian profiles of the few-layered graphene sheets, which are different from the case of few-layered graphene sheets generated by micromechanical cleavage of graphite [11]. The reason is that an ordered stacking (i.e., ABAB stacking) and therefore an electronic coupling do not occur in all region of a CNW sheet [48].

The D peak and 2D' peak are attributed to the structural disorder in the CNW sheets [38]. The intensity of the D band is at least partly a consequence of the high fraction of open edges and pinholes within the CNWs (Figure 2a) [49]. The disorder-induced combination mode (D + G) at about 2,920 cm^-1 ^was also observed. For comparison of the relative intensity of each peak, the Raman spectra were normalized. Both of the G peaks intensities before and after reduction were fixed at 1 (Figure 3a). The band area ratios I(2D)/I(G) increased from 0.79 to 0.81 after thermal reduction. This change indicates a slight increase of sp^2 ^carbon domain. The band area ratios I(D)/I(G) decreased from 1.73 to 1.63 after thermal reduction. The reducing I(D)/I(G) indicates a decreasing degree of disordered carbon. The ratio of the intensity of the G band to that of the D band I(G)/I(D) is directly related to the in-plane crystallite size *L*_a _(nanometers) = 19.2 (I(G)/I(D)), and an increase of *L*_a _from 11.1 to 11.8 nm was obtained [50].

XPS studies reveal the nature of the carbon and oxygen bonds present in the samples (Figure 3b,3c). The XPS peaks were decomposed with a Gaussian fit. Analysis of the CNWs shows a significant reduction of oxygen functional groups after thermal annealing in H_2 _for 2 h at 900°C. Briefly, the as-grown CNWs contained non-oxygenated ring C (71.1%), sp^3 ^C hybridized to C (C-C, 18.5%), C in C-OH bonds (9.1%), the carboxylate carbon (O = C-OH, 1.1%), and carbonyl carbon (<0.2%). After thermal annealing, only a small fraction of C in C-OH (1.7%) remained in the CNWs. C in C = C and C-C bonds increased to 72.8% and 25.5%, respectively. The O1 s spectra showed similar reduction of O - the peak weakened after reduction in H_2 _(Figure 3c). However, the accurate determination of every O-containing group after the thermal reduction is quite challenging due to the insufficient signal-to-noise ratio. Positions of carbon-related and oxygen-related peaks in the XPS spectra are consistent with those of oxidized graphene reported recently [51]. The reduction of oxygen functional groups suggested by the XPS spectra is consistent with the Raman data.

**Figure 4:**
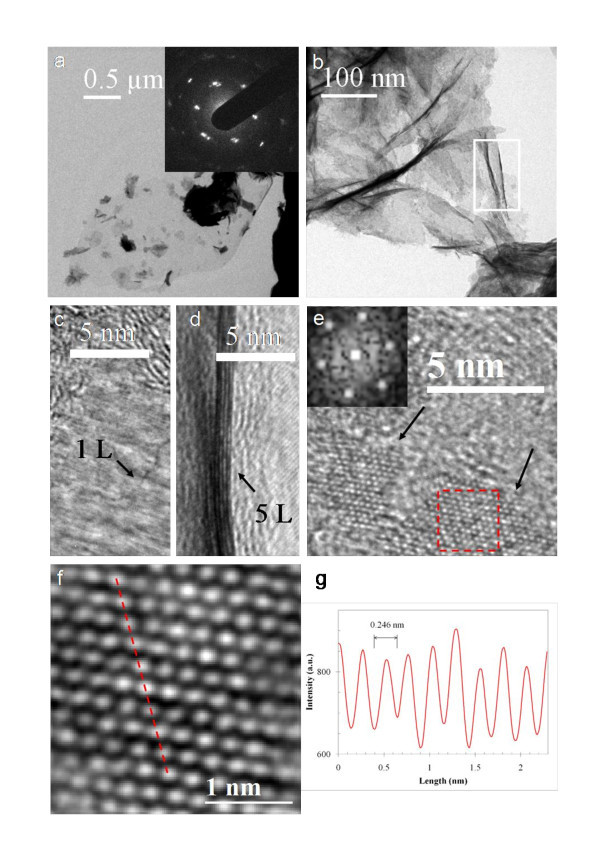
**TEM characterization of CNWs**. **(a) **A CNW sheet supported on a Cu grid. Electron diffraction from the CNW is shown as an inset. **(b) **The areas of a CNW with different thicknesses and wrinkles. **(c) **and **(d) **HRTEM images showing the edges of CNW film consisting of one, and five graphene layers, respectively. (d corresponds to the area defined by the white box in b). **(e) **HRTEM iamge of a CNW sheet with two well-crytallined regions (arrowed). The diffractogram (the inset) is from the red-squared region in (e). **(f) **A filtered image of the squared region in (e). **(g) **The intensity profile along the red dashed line in (f).

TEM images of the product CNWs were shown in Figure 4. Two low-magnification TEM images are shown as Figure 4a and 4b. The inset in Figure 4a is a SAD pattern of the CNW sample, which displays a hexagonal pattern confirming the threefold symmetry of the arrangement of carbon atoms. Well-defined diffraction spots (instead of ring patterns) were observed for most CNWs, while ring patterns were observed seldomly, indicating the mostly few-layer structure and a high degree of crystallinity of the resulting CNWs. HRTEM examination of the samples confirms that the CNW sheets consist of only a few graphene layers (typically one to five layers, Figure 4c,4d). The edges of the suspended CNWs often fold back, allowing for a cross-sectional view of the graphene [48,52]. By observing these edges through HRTEM images, the number of layers at multiple locations on the graphene can be measured (Figure 4c,4d). The estimated interlayer spacing is about 3.50 Å, which is a little larger than the *d*-spacing of graphite (3.36 Å). The small amount of oxygen-containing functional groups might be the main reason for this difference [44].

Although a fraction of surface area of the CNW may be covered with oxygen groups, there are well-crystallined graphitic regions (sp^2 ^carbon) in the CNW. Figure 4e is an HRTEM image from another CNW sample and shows two regions (arrowed) with well-defined fringes implying the good crystallinity of the CNW. The diffractogram (the inset in Figure 4e) of the red-squared region in Figure 4e gives a set of hexagonal spots, suggesting the possible monolayer nature of the region. We further inspected the squared area in Figure 4e by performing Fourier filtering. A filtered image with atomic resolution is shown in Figure 4f. The "honeycomb-like" carbon rings in Figure 4f clearly illustrate that the CNW consists of monolayer graphene. The length of the C-C bond in graphene is 0.142 nm [53], resulting in a hexagon with a width of 0.25 nm. We analyzed the intensity profile (Figure 4g) along the red dashed line in Figure 4e. The hexagon width measured from the intensity outline in Figure 4g is about 0.246 nm, which is in good agreement with the expected value of 0.25 nm. Our HRTEM analysis indicates the existence of monolayer graphene in the product CNWs.

**Figure 5:**
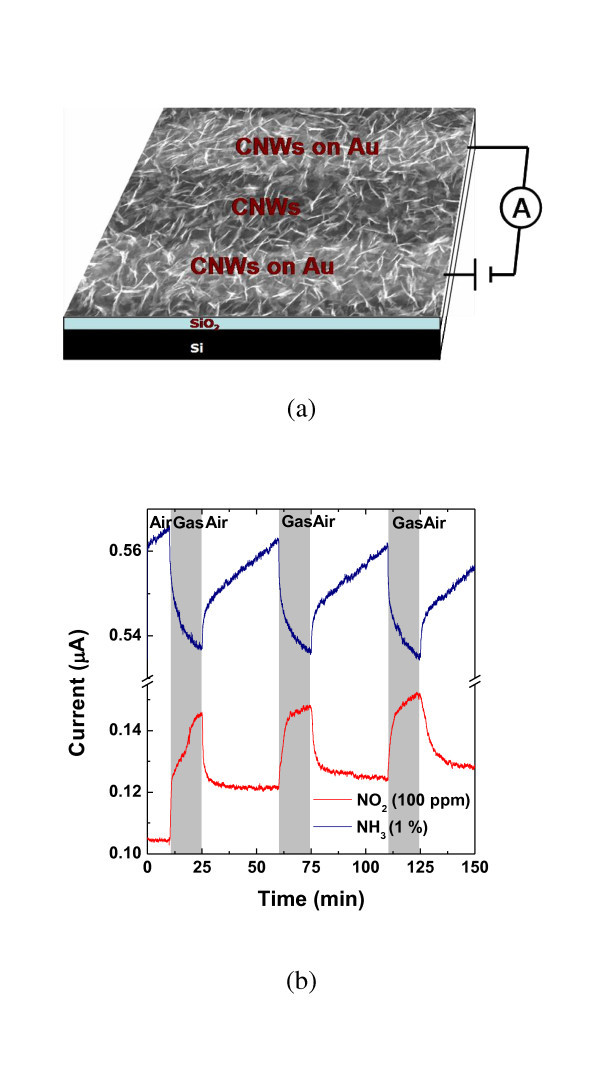
**Gas sensing performance of as-produced CNWs**. **(a) **SEM image of CNWs bridging two neighboring Au fingers of an interdigitated electrode. Gases are detected by measuring the change in the device current while applying a constant dc bias to the device. **(b) **Room-temperature sensing response for NO_2 _and NH_3_.

To demonstrate the gas sensing performance of the as-grown CNWs, CNWs were grown on interdigitated Au electrodes. The interdigitated electrodes with finger width and inter-finger spacing both of 1 μm were fabricated by an e-beam lithography process and used as the sensor substrates [54]. The growth duration was 5 min as it was found that this exposure would yield a CNW film with CNWs connecting with the two neighbouring electrodes (Figure 5a). The sensor operated at room temperature and was periodically exposed to clean dry air flow of 2 lpm for 10 min to record a base value of the sensor conductance, NO_2 _(100 ppm) or NH_3 _(1%) diluted in air of 2 lpm for 15 min to register a sensing signal, and then a lab air flow of 2 lpm again for 25 min to recover the device. A constant dc bias (= 0.1 V) was applied across the two gold terminals.

Upon the introduction of NO_2_, the sensor current went up, i.e., the conductance of the sensor increased (Figure 5b, red curve). Upon exposure to NH_3_, the sensor current went down, i.e., the conductance of the sensor decreased (Figure 5b, blue curve). Thus, the CNW film behaves like a p-type semiconductor, similar to graphene exposed to air. NO_2 _is a strong oxidizer with electron-withdrawing power [55]; therefore, electron transfer from the CNWs to adsorbed NO_2 _leads to increased hole concentration and enhanced electrical conduction in the CNW network. Likewise, the absorbed NH_3 _molecules donate electrons to CNW and neutralize holes partially in the CNW, which results in a lower sensor current in the device. The sensing behavior of the as-grown CNW is consistent with a typical graphene or reduced graphene oxide gas sensor [54].

## Conclusions

In summary, we have demonstrated a new path to low-cost production of CNWs on Si, stainless steel, and Cu substrates with a dc PECVD system operated at atmospheric pressure. SEM, HRTEM, Raman spectroscopy, and XPS reveal that the as-grown CNW material has a significant fraction of chemically functionalized mono- and few-layer graphene, with patches of O-containing functional groups; however, most of the O-containing functional groups can be removed by thermal annealing. Our atmospheric pressure process can be readily scaled up for large area growth through the use of an array of tungsten needle cathodes. A gas sensing device based on as-produced CNW film responds to low-concentration NO_2 _or NH_3 _in a similar fashion as sensing devices based on graphene or reduced graphene oxide. Therefore, a simple one-step gas sensor fabrication process has been demonstrated.

## Competing interests

The authors declare that they have no competing interests.

## Authors' contributions

KHY carried out the CNW synthesis, SEM characterization, growing CNWs into a gas sensor, and drafted the manuscript. ZB provided the basic idea of the dc-plasma reactor design. GHL carried out the TEM and HRTEM characterization, fabricated the sensor electrode, carried out the gas sensing experiments, and helped to draft the manuscript. SM helped to carry out the Raman analysis. SMC carried out the XRD analysis. YWZ participated in the HRTEM characterization. XQC carried out the XPS characterization. RSR and JHC helped draft the manuscript and finalized the version to be published. All authors read and approved the final manuscript.

## Supplementary Material

Additional file 1 **CNWs grown on a Cu plate and stainless steel plates; emission spectrum of dc glow discharge**. Figure S-1 SEM images of CNWs grown on a Cu plate with different surface density. Figure S-2 (a) SEM image showing no presence of CNWs on a stainless steel plate when CH_4 _alone is used as the precursor gas. (b) CNWs grown using CH_4 _and H_2_O. The growth time for both cases is 5 min. Figure S-3 Emission spectrum of glow discharge obtained by subtracting the background signal (without discharge) from the total spectrum (with discharge). Emission lines of OH are remarkable in the spectrum of a CNW sample. Click here for file
