# Supplementary material for: Growth of carbon nanowalls at atmospheric pressure for one-step gas sensor fabrication
Source: Nanoscale Res Lett. 2011 Mar 9;6(1):202. doi: 10.1186/1556-276X-6-202 (PMC3211258; doi:10.1186/1556-276X-6-202)
Supplement: Additional file 1 — CNWs grown on a Cu plate and stainless steel plates; emission spectrum of dc glow discharge. Figure S-1 SEM images of CNWs grown on a Cu plate with different surface density. Figure S-2 (a) SEM image showing no presence of CNWs on a stainless steel plate when CH4 alone is used as the precursor gas. (b) CNWs grown using CH4 and H2O. The growth time for both cases is 5 min. Figure S-3 Emission spectrum of glow discharge obtained by subtracting the background signal (without discharge) from the total spectrum (with discharge). Emission lines of OH are remarkable in the spectrum of a CNW sample. [file 1556-276X-6-202-S1.DOC]

Supplementary Data

Growth of carbon nanowalls at atmospheric pressure for one-step gas sensor fabrication

Kehan Yu, Zheng Bo, Ganhua Lu, Shun Mao, Shumao Cui, Yanwu Zhu, Xinqi Chen, Rodney S. Ruoff, and Junhong Chen


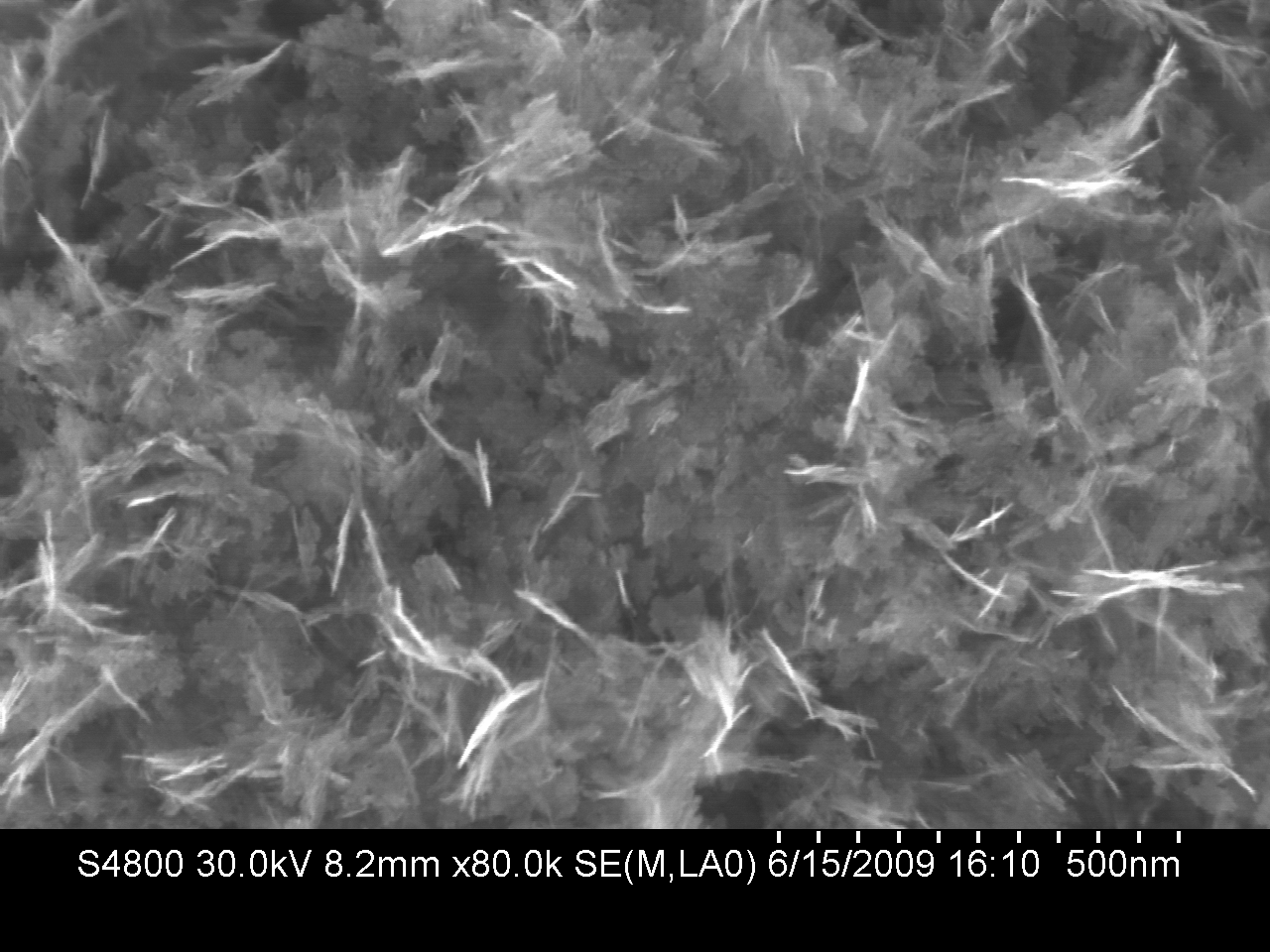


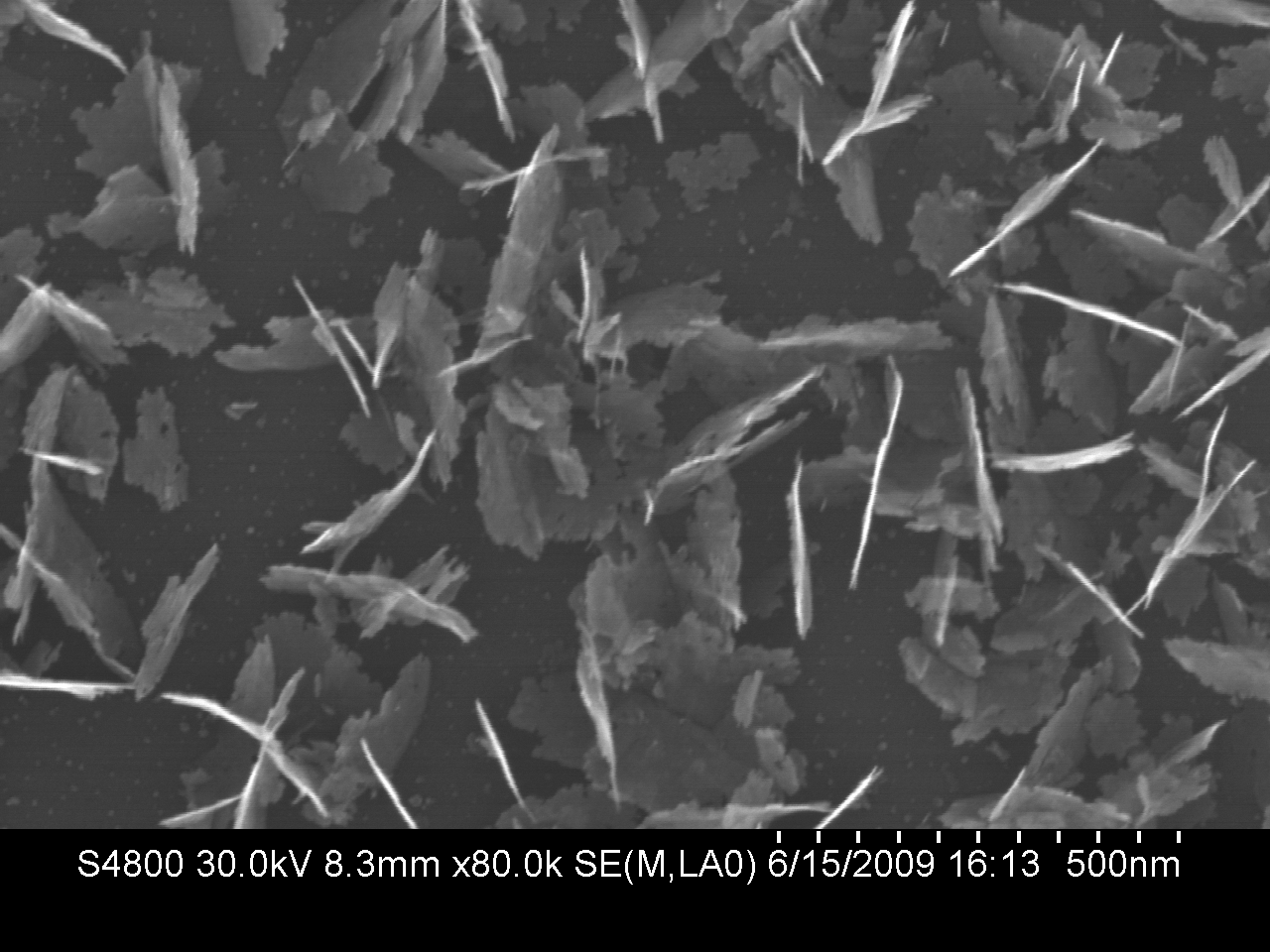


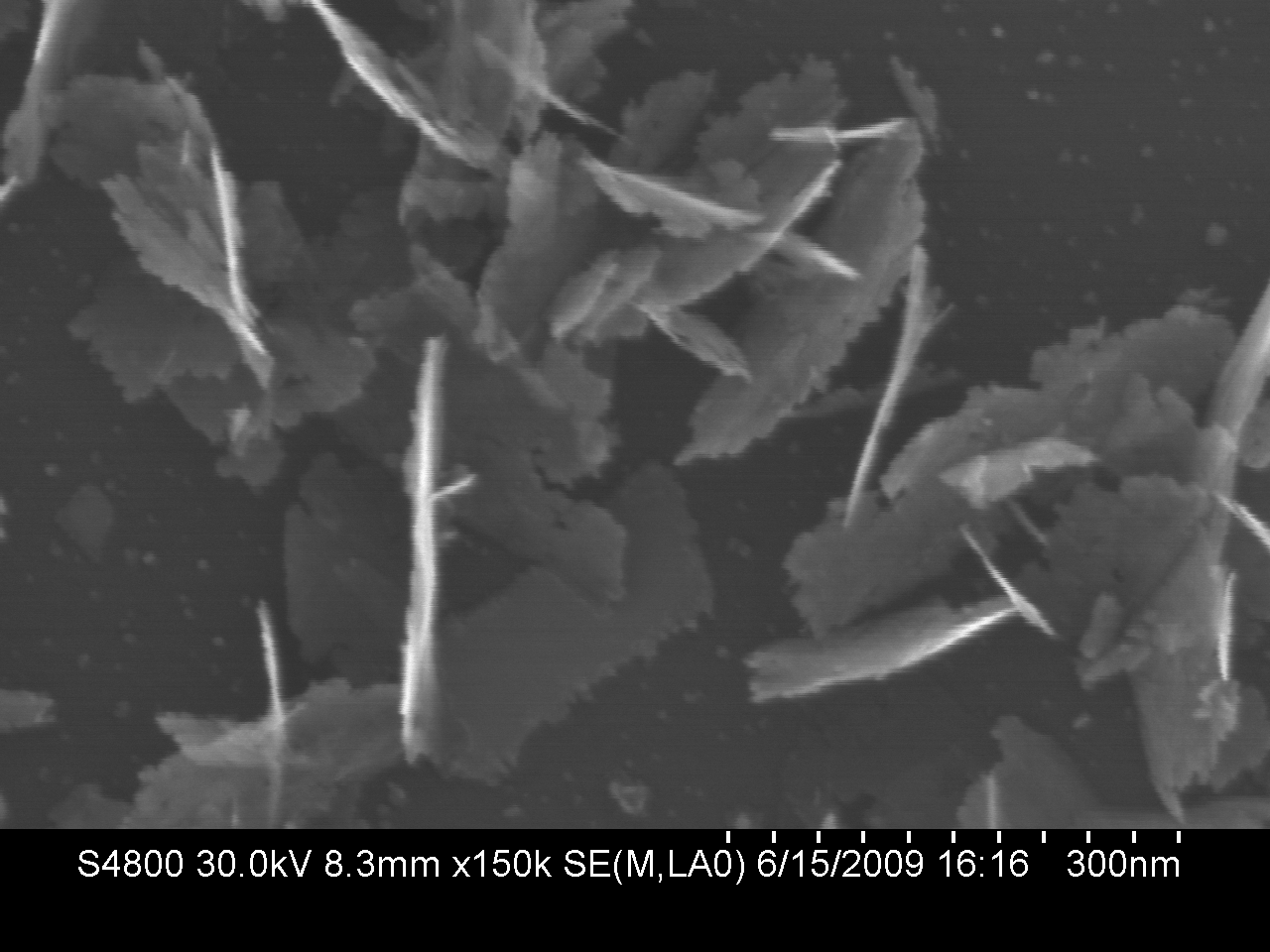


Figure S-1 SEM images of CNWs grown on a Cu plate with different surface density.


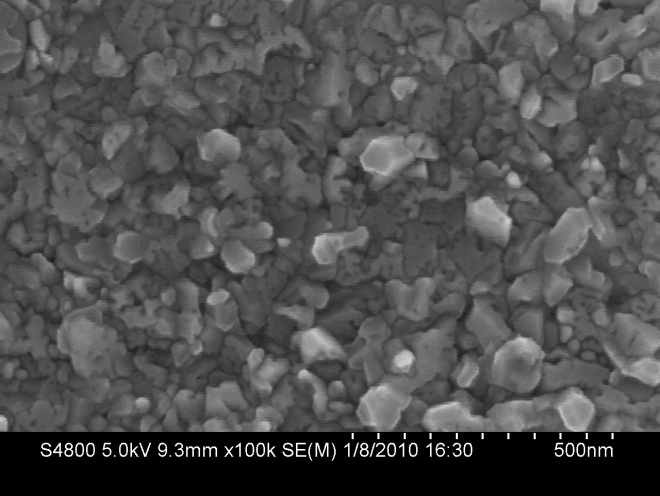


(a)


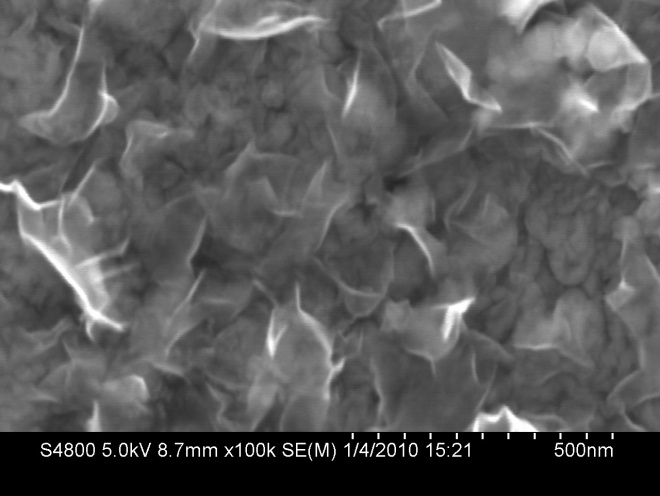


(b)

Figure S-2 (a) SEM image showing no presence of CNWs on a stainless steel plate when CH_4_ alone is used as the precursor gas. (b) CNWs grown using CH_4_ and H_2_O. The growth time for both cases is 5 min.

Figure S-3 Emission spectrum of glow discharge obtained by subtracting the background signal (without discharge) from the total spectrum (with discharge). Emission lines of OH are remarkable in the spectrum of a CNW sample.
